# Breath and plasma metabolomics to assess inflammation in acute stroke

**DOI:** 10.1038/s41598-021-01268-5

**Published:** 2021-11-09

**Authors:** Waqar Ahmed, Iain R. White, Maxim Wilkinson, Craig F. Johnson, Nicholas Rattray, Amit K. Kishore, Royston Goodacre, Craig J. Smith, Stephen J. Fowler

**Affiliations:** 1 Division of Infection, Immunity and Respiratory Medicine, Faculty of Biology, Medicine and Health, University of Manchester, Manchester, UK; 2 Manchester Institute of Biotechnology, University of Manchester, Manchester, UK; 3 Laboratory for Environmental and Life Sciences, University of Nova Gorica, Nova Gorica, Slovenia; 4 Strathclyde Institute of Pharmacy and Biomedical Sciences, University of Strathclyde, Glasgow, UK; 5 Greater Manchester Comprehensive Stroke Centre, Geoffrey Jefferson Brain Research Centre, Salford Royal NHS Foundation Trust, Manchester Academic Health Science Centre, Salford, UK; 6 Division of Cardiovascular Sciences, Lydia Becker Institute of Immunology and Inflammation, Faculty of Biology, Medicine and Health, University of Manchester, Manchester, UK; 7 Department of Biochemistry and Systems Biology, Institute of Systems, Molecular and Integrative Biology, University of Liverpool, Liverpool, UK; 8 NIHR Manchester Biomedical Research Centre, Manchester University Hospitals NHS Foundation Trust, Manchester, UK

**Keywords:** Metabolomics, Neurological disorders, Analytical chemistry

## Abstract

Inflammation is strongly implicated in both injury and repair processes occurring after stroke. In this exploratory study we assessed the feasibility of repeated sampling of exhaled volatile organic compounds and performed an untargeted metabolomic analysis of plasma collected at multiple time periods after stroke. Metabolic profiles were compared with the time course of the inflammatory markers C-reactive protein (CRP) and interleukin-6 (IL-6). Serial breath sampling was well-tolerated by all patients and the measurement appears feasible in this group. We found that exhaled decanal tracks CRP and IL-6 levels post-stroke and correlates with several metabolic pathways associated with a post-stroke inflammatory response. This suggests that measurement of breath and blood metabolites could facilitate development of novel therapeutic and diagnostic strategies. Results are discussed in relation to the utility of breath analysis in stroke care, such as in monitoring recovery and complications including stroke associated infection.

## Introduction

Stroke is the second leading cause of death worldwide^1^ and the leading cause of adult neurological disability^2^. The onset of cerebral ischaemia induces an inflammatory response in the central nervous system (CNS) and periphery, characterised by activation of resident CNS immune cells, recruitment of peripheral leucocyte populations and a systemic acute phase response^3^. Inflammation is strongly implicated in both the injury and repair processes occurring after stroke and may represent opportunities for therapeutics. Numerous inflammatory mediators in the peripheral circulation, such as C-reactive protein (CRP) and interleukin-6 (IL-6), are altered in the acute phase of stroke and could have a role as biomarkers of prognosis or impending complications^4,5^.

Advances in sampling and analytical methods have led to the successful application of metabolomics in several inflammatory diseases^6,7^. Biomarkers that reflect disruption of metabolic pathways after stroke could facilitate development of novel therapeutic and diagnostic strategies^8^. An alternative non-invasive approach to monitoring post-stroke complications is to analyse exhaled volatile organic compounds (VOCs). Breath analysis offers a biologically relevant systems-approach, which has shown promise in detecting relevant airway pathogens in mechanically ventilated patients^9–11^. Serial breath sampling in non-ventilated acute stroke patients is however potentially challenging, as altered awareness, cognitive and language difficulties, impaired sitting balance and hemi-facial weakness are frequently encountered.

Infections are the most common complication of stroke and exacerbate the inflammatory response^12^. Pneumonia is the most frequent infection, occurring in around 14% of patients, and as many as 35% of those at highest risk^13^, and independently predicts death and worse outcomes in survivors^14^. At present, existing blood biomarkers add little to clinical prediction algorithms^15^, and development of biomarkers to enable early detection of pneumonia and response to antibiotic treatment would have considerable value in stroke care and antibiotic stewardship. A range of VOCs have been identified in previous studies as potential markers of infection^9,16^ and inflammation^17,18^, and could therefore have value in the setting of acute stroke.

We therefore conducted a study to assess the feasibility of combined breath and plasma sampling for metabolomic analysis in patients admitted to hospital after acute stroke. The primary objective was to refine our existing breath sampler methodology for use in the acute stroke setting and establish the feasibility of alternate-day breath sampling during a relevant timeframe after admission. A secondary objective was to explore contemporaneous exhaled breath, plasma metabolomic and cytokine profiles in patients considered at high-risk of developing pneumonia.

## Methods

### Study assessment schedule

The study schedule was based on the course of the most frequently encountered complications of stroke. Infections, particularly pneumonia, occur most frequently within the first week after stroke onset, and antibiotic treatment is typically prescribed for at least 5–7 days. Our breath sampling schedule was therefore designed to evaluate the feasibility of repeated measurements starting within 24 h of symptom onset, and on alternate days to day 15 (or discharge from inpatient stroke services if this occurred sooner). Each participant underwent a maximum of eight scheduled breath sampling and clinical assessments. This schedule would be appropriate aimed obtain breath measurements prior to the clinical manifestation of pneumonia, during clinical investigation of suspected pneumonia, and both during and after antibiotic therapy. This timeframe also incorporates the peak in levels of inflammatory mediators.

### Study participants

Patients with ischaemic stroke or intracerebral haemorrhage (ICH) presenting to the Comprehensive Stroke Centre (CSC) at Salford Royal Hospital within 24 h of the onset of stroke symptoms were screened for inclusion. In order to assess the risk of developing pneumonia, we used the A^2^DS^2^ score, a 10 point clinical score based on age, presence or absence of atrial fibrillation, dysphagia, sex and stroke severity^19^. The A^2^DS^2^ score is an externally validated risk score in stroke patients with good performance metrics^20^. Patients aged over 17 years, with an ischaemic stroke or ICH and an A^2^DS^2^ score of ≥ 3 were eligible. Informed consent was provided by participants, or by a personal consultee in situations where capacity was lacking. Approvals were obtained from the Betsi Cadwaladr University Health Board regional ethics committee (Ref: 14/WA/0018) and Trust Research and Development department. All research methods were performed in accordance with the relevant guidelines and regulations. The CSC at Salford Royal operates a hub and spoke model, and patients residing outside the local area who present to the CSC in the hyperacute or acute phase are repatriated to their base hospital after completion of an acute care bundle. Therefore, only patients with a Salford area postcode were considered for screening (to avoid early repatriation of participants to their base hospital). Patients were additionally excluded for any of the following reasons: (1) informed consent was not possible; (2) rapidly improving symptoms at the time of screening; (3) infection treated with antibiotics in the preceding 6 weeks; (4) mechanical ventilation or palliative care considered imminent. A secure log was kept of all patients screened for the study, including the reason for non‐inclusion.

### Clinical assessments

Baseline stroke subtype (ischaemic stroke or ICH), stroke severity using the National Institutes of Health Stroke Scale (NIHSS)^21^ score, swallowing status, vascular risk factors, medications, independence pre-stroke using the modified Rankin score (mRS)^22^ and vital signs were recorded at study entry. In addition, swallowing and feeding status, oxygen requirements, vital signs, standardised infection screening log, antibiotic prescriptions, microbiology data, chest radiography results and any antibiotic prescriptions were recorded by the study team on each scheduled study assessment. Clinician-diagnosed and treated infections were adjudicated by the study team using the Centers for Disease Control and Prevention (CDC) criteria^23^. End of study mRS was recorded at day 15, or at discharge if this occurred earlier.

### Development of the breath sampler for stroke unit setting

The sampler was designed as a portable system capable of supplying VOC-filtered air to a breathing mask, providing constant airflow. The method and sampler components were based on our previous sampler^24^ but the integration of new components resulted in a smaller footprint such that the complete system along with accessories and laptop could be transported to the bedside on a standard hospital trolley. Briefly, air is supplied through a one-way non re-breathing valve to a mask (ResMed, Mirage Full Mask Series 227, Resmed, Abington, UK) via a VOC filter (AX:456-06-20, 3M, Bracknell, UK) and an adapted CPAP machine (Resmed Stellar, Resmed). Pressure sensors at the patient interface allow tracking of the breathing cycle and enable targeted sampling of the late expiratory phase even with variable breathing patterns. During sampling, breath is diverted through a 3.5″ stainless steel tube containing a dual bed of sorbent material for VOC capture (Carbograph 2TD 40/60 mesh, Carbograph 5TD 40/60 mesh; Markes International, UK) using a diaphragm pump (Escort ELF Pump, MSA) at a flow rate of 1 L min^−1^. A picture of the breath sampler is shown in supplementary material, Fig. S1.

### Breath sampling and analyses

All breath sampling was undertaken by stroke research nurses trained and assessed in the sampling procedure. After each participant was fitted with the mask they were allowed to acclimatise to the system for 10 min prior to breath collection. Approximately 2 L of late-expiratory breath was sampled through each sorbent tube. To minimise the potential impact of diurnal variation^25^, all breath sampling after study entry was between 07:00 and 11:30. Following breath sampling, tubes were immediately sealed and stored at 4 °C until the time of their analysis.

A gaseous standard (100 μL of 1 ppmV 4-bromofluorobenzene in nitrogen; Thames Restek, UK) was added to each sorbent tube as an internal standard prior to analysis by thermal desorption-gas chromatography-time of flight-mass spectrometry (TD-GC-ToF-MS). Tubes were thermally desorbed at 280 °C for 5 min in 50 mL min^−1^ of carrier gas and cryo-focussed onto a cold trap maintained at 10 °C. Analytes were then desorbed from the cold trap at 280 °C for 3 min and transferred to a GC oven (6890 series, Agilent, UK) via a heated transfer line maintained at 200 °C. Flow to the column (DB-5MS, 30 m, 0.25 mm I.D., 0.25 μm film thickness, Agilent) was maintained at 1 mL min^−1^ and the oven was set to an initial temperature of 40 °C (no hold time), with a primary ramp to 170 °C at 6 °C min^−1^ and a secondary ramp to 190 °C at 15 °C min^−1^ with a total run of 23 min (followed by a 2 min post-run at 250 °C). Analytes were detected by time of flight mass spectrometry (Micromass GCT Premier, Waters, UK) with an electron ionisation source at 70 eV. The source temperature was set to 200 °C, at a trap current of 200 μA and spectra were recorded in dynamic range extension mode at 10 Hz over a range of 50–650 m/*z*. The [C_4_F_9_]^+^ fragment of heptacosafluorotributylamine was used as a lock mass (218.9856 Da). Samples were analysed in random order and each patient sample was analysed alongside a 24-component external standard^26^. A further 22 compounds, selected from literature as markers of infection and inflammation, were included in a target list and peak areas were extracted based on accurate mass fragments (Table S1).

The following quality control criteria were adopted to ensure exhaled VOCs could be precisely quantified: 1. Internal standard parent ions (*m*/*z* 173.9480 and 175.9460) did not deviate by more than 5% within a batch, in abundance or ratio (to ensure that any water remaining on the tubes after their pre-purge had not affected analyses); 2. If the sum total-ion-count of a chromatogram (once background subtracted for the lock-mass compound) deviated by more than 10% within in a batch, this run was flagged. These samples were then rejected from further data analysis if dodecamethylcyclohexasiloxane was not detected (at *m*/*z* = 429 at a retention time of 15.90 min). None of the samples were rejected due to water retention however several were discarded due to low VOC recovery, most likely due to the irreversible sorption of semi-volatile compounds onto the strongest sorbent following inadvertent sampling in reverse orientation. Whilst simple strategies can be adopted to abate this problem, it is only apparent after thermal desorption and highlights an important consideration for future studies given that multi-bed sorbent tubes are currently a common choice in breath research^27^.

### Blood sampling and analyses

Venous blood was drawn at study entry, day 3, day 5–7 and day 15 for analysis of plasma metabolomics, IL-6, CRP, total white blood-cell (WBC) count and automated differential leukocyte counts. Blood draw was standardised between 07:00 and 11:00, except for study entry. For plasma analyses, blood was collected in 4.5 mL heparinised tubes (for metabolomics) and 6 mL EDTA (for CRP and IL-6), centrifuged at 2000×*g* at 4 °C for 15 min, and frozen at − 70 °C. An additional 3–4 mL of blood was collected in EDTA for total WBC and automated differential using the Coulter principle.

Plasma IL-6 was measured using Luminex bead technology (Luminex, Austin, TX, USA). Bio-Plex COOH beads (Bio-Rad Laboratories, Hemel Hempstead, UK) were coupled to Pelikine anti-IL-6 monoclonal antibodies (Cat: M191602, MAST Group Ltd, Bootle, UK). The detection antibody was Pelikine anti-IL-6 (Cat: M191604) and 15% horse serum, 5% bovine serum, and 1% mouse serum in high performance ELISA buffer (HPE; Pelikine) was used as diluent. Plasma CRP was measured in a single-plex competitive assay using Luminex bead technology (Luminex, Austin, TX, USA). Bio-Plex magnetic COOH beads (Bio-Rad Laboratories, Hemel Hempstead, UK) were coupled to Biodesign anti-CRP monoclonal antibody (cat: M86842M, clone C2). The competitor was CRP (P100-0; SCIPAC, Sittingbourne, UK) biotinylated with Pierce EZ-Link Sulfo-NHS-LC-LC-Biotin (Pierce, Rockford, IL, USA). 10% horse serum, 5% bovine serum, and 1% mouse serum in tris-buffered saline was used as diluent. Binding of biotinylated CRP or IL-6 detection antibody was assessed, following addition of R-Phycoerythrin Streptavidin (Jackson ImmunoResearch Laboratories Inc, Stratech, Newmarket, UK; Cat: 016-110-084), using a Bio-Plex 200 system. The assays were calibrated against current National Institute for Biological Standards and Control standards (NIBSC, South Mimms, UK) standards.

Plasma metabolomic analysis was performed using ultra-high performance liquid chromatography-mass spectrometry (UHPLC-MS). Samples were lyophilised by drying the supernatant from protein precipitation with LC-MS grade methanol (1:4 ratio plasma:methanol). Lyophilised sample pellets were reconstituted in LC-MS grade water (100 μL) and added to LC-MS clear vials with fixed inserts. Analysis was carried out on an Accela UHPLC auto-sampler coupled to an electrospray LTQ-Orbitrap XL hybrid mass spectrometer (Thermo Fisher, Bremen, Germany), following established protocols^28^. Xcalibur and TunePlus software (version 2.0, Thermo Fisher, Bremen, Germany) were used for instrument operation. Tuning and calibration was carried out as per the manufacturer's instruction. Samples were analysed in a random order whereby 5 μL aliquots were injected onto a Hypersil Gold C18 reversed phase column (100 mm × 2.1 mm × 1.9 μm) and a methanol/water solvent gradient was used to separate metabolites on the stationary phase (95% water 5% methanol for solvent A and 5% water 95% methanol for solvent B). Both solvents contained 0.1% formic acid to aid ionization within the source. Samples were analysed in positive ESI mode using the following settings: 1 micro scan per 400 ms, 50–2000 m/*z* range, source transfer tube set at 275 °C, tube lens voltage set at 100 V, capillary voltage set at 30 V, sheath gas flow rate set at 40 arbitrary units, auxiliary gas flow set at 5 arbitrary units and sweep gas at 1 arbitrary units. Data were collected in profile mode at a mass resolution of 30,000.

### Breath sampling feasibility

The feasibility of the breath sampling protocol was evaluated by recording the proportion of eligible patients declining participation (and reasons given), time taken to undertake breath sampling, and the proportion of participants completing alternate day breath sampling from study entry to end-of study as per sampling protocol. Sampling reliability was assessed by calculating intraclass correlation coefficients (two way) across selected timepoints (n = 4) for patients without missing visits (n = 5). An ICC of less than 0.5 was interpreted as poor, between 0.5 and 0.74 as moderate, and greater than 0.75 as good. Negative ICC values were interpreted as 0. The tolerability of serial breath analysis was assessed as the proportion of participants unable to complete the breath sampling protocol on one or more occasions (and reasons given if not completed). Data were described using appropriate summary statistics. We planned additional exploratory secondary analyses to investigate the presence of previously published pathogen-associated metabolites detected in exhaled breath, plasma or both. As this was a feasibility study, a power calculation was not performed. Instead we estimated that recruitment of at least 20 patients meeting the eligibility criteria would enable assessment of the primary aims of feasibility and tolerability.

**Figure 1:**
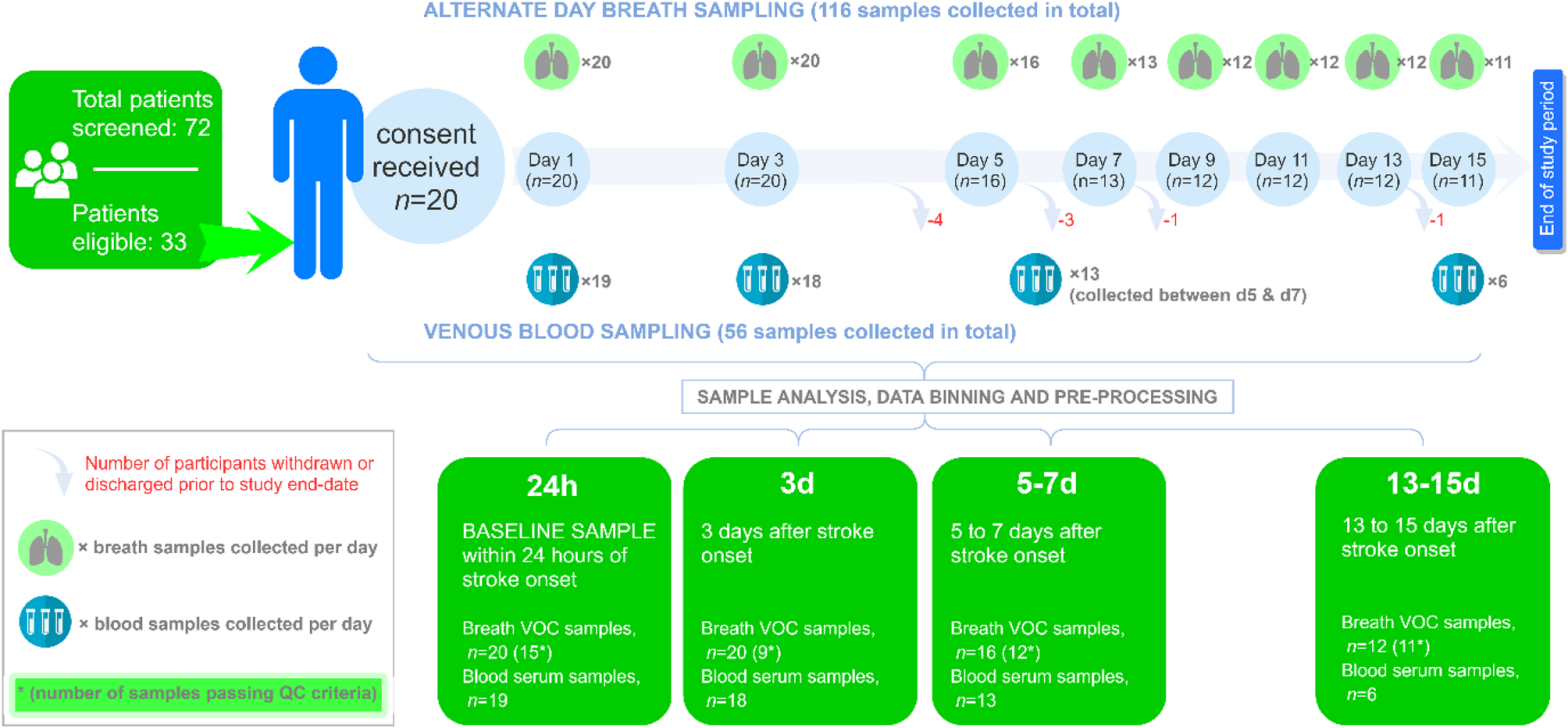
Study assessment overview depicting the type and number of samples collected.

In order to compare the progression of VOC profiles alongside blood metabolites and inflammatory cytokines post-stroke onset, data were binned into four time-periods as depicted in Fig. 1.

### Data pre-processing and treatment

Blood plasma sample data were converted to netCDF format and pre-processed using the XCMS Centwave peak picking algorithm^29^ and LOESS retention time correction. Pooled QC sample data were used to align instrument drift through Robust Spline Correction^30^. Data were then treated by log_10_ transformation and autoscaling. For exhaled VOCs, selected ion peak areas were extracted and normalised across all breath samples using TargetLynx software (Masslynx version 4.1, Waters Corporation, UK). Non-detected VOC peak areas (total *n* = 108) were treated as missing values and excluded from statistical tests. The R packages employed in this study were *xcms*^31^, *corrplot*, *irr*, *tidyverse*, and *adegenet*^32^.

### Statistical analysis

Univariate and multivariate statistical analyses were performed in R (version 3.5) and Graphpad Prism (version 7.03). The non-parametric multiple comparisons Kruskal–Wallis test was used to screen breath VOCs and inflammatory markers across timepoint samples by comparing mean peak areas^33^. For significant differences, a post-hoc Dunn’s test was used to further explore change in peak area between two groups. False discovery rate was used to adjust for multiple comparisons in all other analyses. To model discriminatory plasma features based on post-stroke sampling time, supervised classification Principal Component-Discriminant Function Analysis (PC-DFA) was carried out; this maximised inter-group variation whilst minimising intra-group variation^34^. Model parameters (number of PCs = 15, and number of discriminant functions = 4) were cross-validated using stratified random sampling (× 1000 sample replications). Pearson’s *r* was used to evaluate correlations between inflammatory markers, VOCs and plasma metabolites.

### Metabolite identification

Identification of selected VOC compounds was confirmed using chemical standards in agreement with the metabolomics standards initiative for chemical analysis^35,36^. Specifically, VOCs with two orthogonal means of identification (i.e. chemical standards and mass spectra library search (NIST 05)) were annotated as level 1, whereas identification solely based on a mass spectra library search and retention index were classified as level 2.

All plasma metabolites were annotated as MSI level 2 as identification was based on accurate mass data. Plasma metabolite identification was predicted through molecular networking using the online version of *Mummichog*^37^. Each feature from pre-processed data was subjected to a Kruskal Wallis test to assess differences between the mean of timepoint groups. The resulting list of m/z values, retention times, and FDR adjusted *p* values was used as input for molecular network analysis. Thresholds of m/z differences were set to 10 ppm. All features were annotated with positive ion mode adducts such as [M + H] + and [M + Na] + . Using these data, features are annotated with an Empirical identification (EID) based on concerted metabolite activities, which are clustered based on similarities and mapped to known metabolic pathways and ordered based on significance through a permutation test (α < 0.05). Cytoscape 3.4.0^38^ was used to visualise network maps.

## Results

### Screening, recruitment and participant characteristics

During the study recruitment period (8 September 2014 to 31 March 2016), we screened 72 patients, of whom 33 were eligible. Of these, informed consent was obtained for 20 participants (Fig. 1). Of the remaining 13 patients, nine declined participation (six due to lack of interest in the study and three due to lack of interest in research participation in general) and it was not possible to obtain informed consent directly from the patient (and a consultee was not available) in the other four.

The baseline characteristics of the 20 participants are shown in Table 1**.** The majority of patients were dysphagic at study entry with moderate severity stroke. Four of the 19 patients (21%) with ischaemic stroke received intravenous thrombolysis. There were no physician-diagnosed or CDC-confirmed episodes of pneumonia during the period of breath sampling. There were six CDC-confirmed urinary-tract infections (UTIs) in five participants occurring at a median (min, max) of 9 (3, 15) d.

**Table 1:** Baseline characteristics of the participating patients.

Total number of participants, *n*	20
Age/[years]	71.7 ± 14.5
Male sex, *n* (%)	12 (60)
National Institutes of Health Stroke Scale score	9.5 (2, 28)
**Hemisphere, *n*** **(%)**	
Left	9 (45)
Right	9 (45)
Bilateral	2 (10)
**Stroke type,** ***n* (%)**	
Ischaemic	19 (95)
Total anterior circulation infarct	10 (50)
Partial anterior circulation infarct	4 (20)
Posterior circulation infarct	3 (15)
Lacunar infarct	2 (10)
Intracerebral haemorrhage	1 (5)
Glasgow Coma Scale score	14 (9, 15)
A^2^DS^2^ score	6 (3, 10)
Nil by mouth, *n* (%)	12 (60)
**Vascular risk factors, *n*** **(%)**	
Hypertension	15 (75)
Diabetes mellitus	2 (10)
Previous stroke or transient ischaemic attack	5 (25)
Coronary artery disease	7 (35)
Atrial fibrillation	9 (45)
Hyperlipidaemia	11 (55)
Current smoker	4 (20)
Preceding modified Rankin score	0 (0, 4)
Total white blood cell count/[10^9^ L^−1^]	9.2 (6.7, 11.8)
Plasma C-reactive protein/[mg L^−1^]	4.8 (0.1, 26.2)
Plasma interleukin-6/[pg mL^−1^]	6.0 (2.6, 40.5)

Data shown as mean ± SD or median (min, max) except where indicated; A^2^DS^2^ score: Age, Atrial Fibrillation, Dysphagia, Sex, stroke Severity.

### Feasibility and tolerability of serial breath sampling

The median (min, max) time from stroke symptom onset to first breath sampling was 16.5 (6.0, 23.8) h. Of the 20 participants, 11 (55%) remained inpatients at the CSC at least to their day 15 assessment, completing all 8 scheduled assessments. The median (min, max) end-of study mRS was 4 (0, 5). One participant deteriorated prior to their day 5 assessment, requiring intubation and ventilation due to significant bulbar involvement and retained respiratory secretions (but no subsequent confirmed pneumonia) and was withdrawn from the study by the research team. The remaining 8 participants were discharged from the CSC prior to the day 15 assessment, but after completing the day 3 (3 participants), day 5 (3 participants), day 7 (1 participant) and day 13 (1 participant) assessments with breath sampling. Therefore, in total 116 breath samples were collected from the 20 participants; all sampling episodes were well-tolerated and completed successfully. Overall, the median (min, max) number of breath samples per participant was 8 (2, 8). During the study, 8 participants (40%) had a nasogastric tube in situ and 4 (20%) others had supplemental oxygen provided via nasal cannulae on at least one occasion during breath sampling. The median (min, max) duration of breath sampling at the bedside was 6 (3, 30) min. Intraclass correlation coefficients showed good intra-patient reliability for VOCs commonly found in breath samples and poor ICC for those VOCs thought to have an environmental origin (Table S1).

### Inflammatory mediators

Differential neutrophils were found to strongly correlate with total WCC (*r* (n = 47) = 0.90, *p* < 0.001) and weakly correlated with differential monocytes (*r* (n = 47) = 0.68, *p* < 0.001). Furthermore, the time-averaged (median) neutrophil concentration calculated per patient weakly correlated with stroke severity (NIHSS score; *r* (n = 20) = 0.72, *p* < 0.001). No significant correlations were found between inflammatory markers and stroke severity or A^2^DS^2^ score based on either levels obtained within 24 h of stroke onset, time-averaged concentrations or peak concentrations. However, peak plasma concentrations of CRP were found to correlate with peak levels of IL-6 (*r* (n = 20) = 0.78, *p* < 0.001).

**Figure 2:**
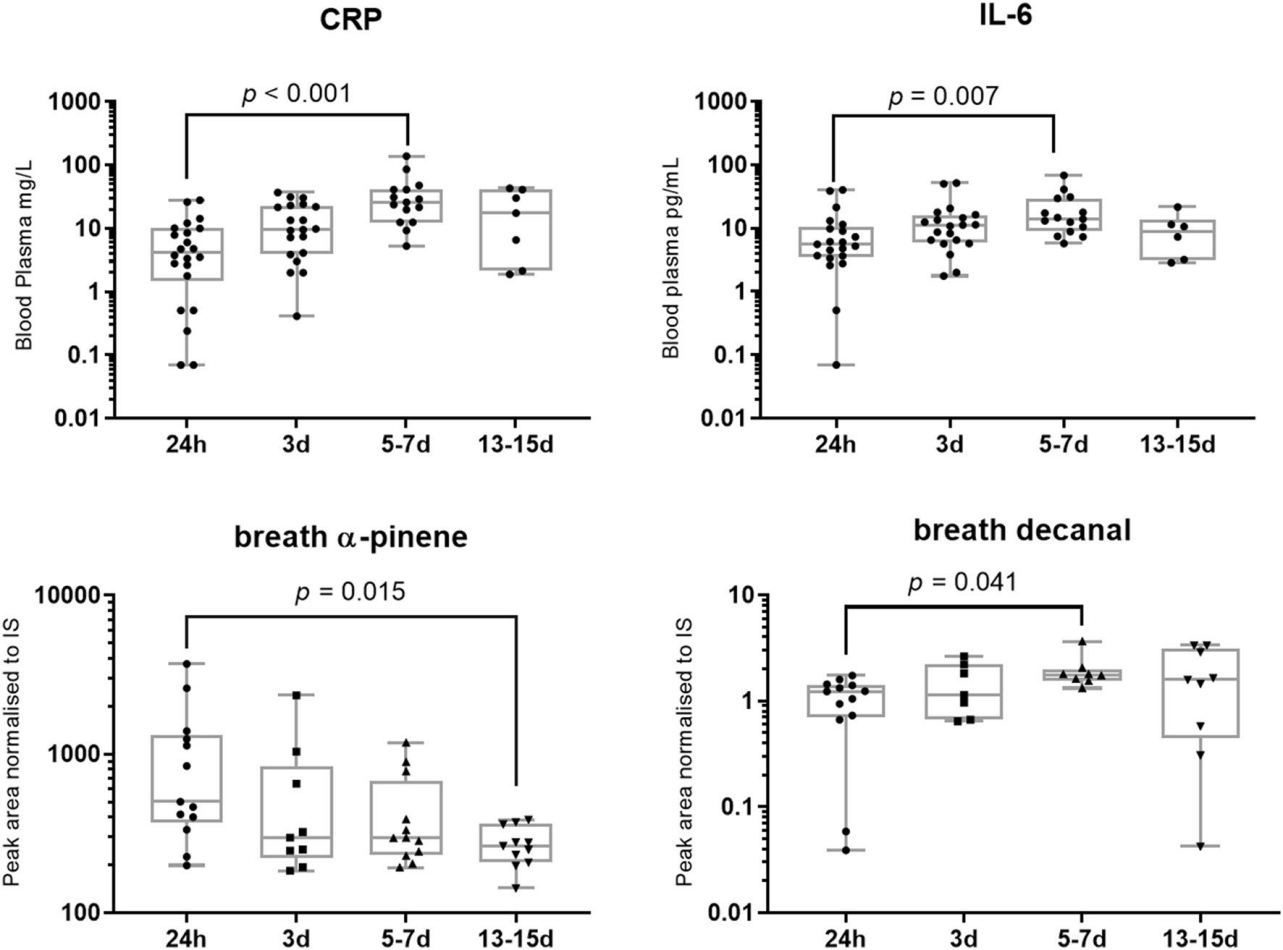
Boxplots (with median line, and whiskers of mininum and maximum value) annotated with Dunn’s test *p *values showing timepoint group differences for CRP concentration (top left, 24 h = 19; 3 days = 18; 5–7 days = 13; 13-15 days = 6), IL-6 concentration (top right, 24 h = 19; 3 days = 18; 5–7 days = 13; 13–15 days = 6), breath α-pinene peak area (bottom left, 24 h = 13; 3 days = 9; 5–7 days = 12; 13–15 days = 11), and breath decanal peak area (bottom right, 24 h = 13; 3 days = 7; 5–7 days = 8; 13–15 days = 9). IS: internal standard.

The inflammatory mediators CRP and IL-6 changed significantly over time (CRP *H* = 19.29, *p* < 0.001; IL-6 *H* = 11.02, *p* = 0.012, Fig. 2). *Post-hoc* testing confirmed the difference between the means of the 24 h and 5–7 days timepoints (CRP *p* < 0.0001, IL-6 *p* = 0.007).

### Breath metabolites

Table S1 shows details of the 46 VOCs that were targeted across breath samples (*n* = 47), representing multiple chemical classes. There was a significant difference between the 24 h and 13–15 days timepoints for α-pinene (*p* = 0.015) and between 24 h and 5–7 days for decanal (*p* = 0.041), as shown in Fig. 2. A paired t-test confirmed the significant change in abundance for decanal in patients sampled at both 24 h and 5–7 days timepoints (*n* = 8, *t* = 4.012, *df* = 5, *p* = 0.0007). Results for all breath VOCs are summarised in Table S2.

### Blood plasma metabolites

Plasma metabolite sample (*n* = 56) data processing generated 2420 mass spectral features (individual ion intensities annotated with *m*/*z* and retention time values). Molecular networking was used to identify compounds which drove temporal variation between blood plasma metabolites. Out of 2420 features, 357 could be mapped to empirical IDs (tentative metabolite IDs) and 112 were selected based on the significance between timepoints (*p* < 0.05). This is visualised in Manhattan plots (supplementary Fig. S2). The network map in supplementary Fig. S3 illustrates clustered groups with concerted metabolite activities. Of the 112 features significantly different between timepoints, 11 features with empirical IDs (equivalent to MSI level 2, i.e., accurate mass only) were mapped to metabolic pathways after a permutation test (*p* < 0.05). Table 2 shows the top metabolic pathways ordered by significance and the empirical IDs associated with each metabolic pathway. Molecular networking provided two possible identities for the significant feature 176.0315 × 1.37: monodehydroascorbate and glucurono-6,3-lactone. The features that were linked to significant empirical IDs and likely metabolite candidates are listed in Table 3.

**Table 2:** Top metabolite pathways associated with significant differences between timepoints.

Pathways	Pathway size	*p* value	EID overlap
Carnitine shuttle	20	0.00025	E18,E83,E286,E165,E68,E258
Saturated fatty acids β-oxidation	2	0.00084	E286,E49
D4&E4-neuroprostanes formation	4	0.00462	E96,E311
Ascorbate (Vitamin C) and Aldarate Metabolism	8	0.02101	E96,E311
3-oxo-10R-octadecatrienoate β-oxidation	8	0.02101	E189, E304
Purine metabolism	9	0.02849	E96, E311
Phosphatidylinositol phosphate metabolism	2	0.03496	E49

EID , Empirical ID; Pathway size , number of features associated with each pathway; EID overlap , features within the pathway which are significant.

**Table 3:** List of features with metabolite identifications from molecular networking analysis, with Pearson’s correlation coefficients for inflammatory markers and breath decanal, and PC-DFA loadings.

Feature		Predicted metabolite identification (MSI level 2)				Pearson's *r*				Timepoint – KW	
ID (*m*/*z* × RT/[min])		Empirical ID	Compound name	Adduct	Mass error/[ppm]	CRP	IL-6	Decanal	α-pinene	Statistic	FDR-adjusted *p* value
371.3022 × 11.10		E165, E83	Tetradecanoyl carnitine	[M]^+^	3.8	–	–	− 0.38 (*p* = 0.036)	–	18.8	0.018
372.3142 × 11.50				[M + H]^+^	9.1	− 0.33 (*p* = 0.016)	–	− 0.40 (*p* = 0.027)	–	16.3	0.035
427.3657 × 12.20		E68	Stearoyl carnitine	[M]^+^	1.2	–	–	− 0.36 (*p* = 0.044)	–	15.8	0.039
502.3846 × 13.10		E18	Tetracosapentaenoyl carnitine	[M + H]^+^	9.0	0.31 (*p* = 0.025)	0.33 *(p* = 0.016)	0.36 (*p* = 0.049)	–	16.0	0.039
501.3815 × 13.11				[M]^+^	0.6	0.39 (*p* = 0.003)	0.35 (*p* = 0.008)	–	–	5.8	0.342
399.3339 × 11.69		E286	Palmitoyl carnitine	[M]^+^	2.5	–	–	–	–	19.8	0.015
526.3817 × 13.49		E258	Tetracosatetraenoyl carnitine	[M + Na]^+^	9.5	− 0.41 (*p* = 0.002)	− 0.41 (*p* = 0.002)	–	–	21.3	0.008
256.2378 × 11.80		E49	Hexadecanoate (n-C16:0)	[M]^+^	9.4	− 0.27 (*p* = 0.049)	− 0.29 (*p* = 0.033)	–	–	21.3	0.015
176.0315 × 1.37		E96	Monodehydroascorbate	[M + H]^+^	0.6	–	–	–	–	16.2	0.035
		E311	Glucurono-6,3-lactone	[M]^+^	3.4					16.2	0.035
265.1801 × 11.76		E304	8-Hydroxy-hexadeca-2E,6E,10Z-trienoate	[M]^+^	1.1	–	–	–	–	23.5	0.008
237.1482 × 10.78		E189	6-Hydroxy-tetradeca-2E,4E,8Z-trienoate	[M]^+^	3.8	–	–	− 0.48 (*p* = 0.007)	–	28.3	0.003

**Figure 3:**
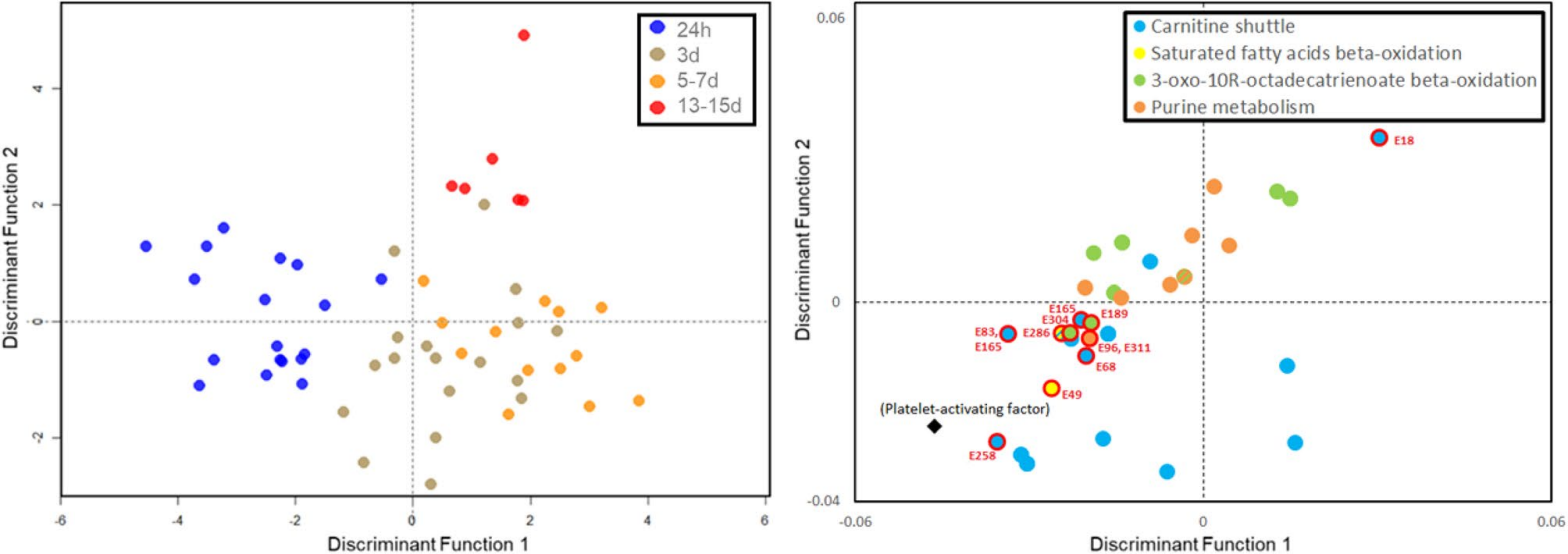
(Left) PC-DFA scores plot with blood plasma samples (*n* = 56) coloured by timepoint groups and (right) loadings plot showing EIDs from the molecular network analysis coloured by biochemical pathway. The significant EIDs listed in Table 3 are encircled in red. Details of all EIDs are listed in Table S3.

A PC-DFA classification model used 2 discriminant functions and 15 PCs selected from model parameter cross validation based on the 4 timepoint groups (24 h, 3 days, 5–7 days, and 13–15 days). The PC-DFA scores plot (the left-hand side of Fig. 3) illustrates a shift through timepoints: 24 h, 3 days and 5–7 days on the first discriminant function, whilst the second discriminant function separates timepoint 13–5 days from the first three.

Presented on the right-hand side of Fig. 3 are the loadings for EIDs associated with the molecular network analysis. Metabolites associated with specific biochemical pathways appear to cluster in regions of the loadings plot, suggesting similar patterns in time profiles for these metabolites. In general, metabolites with negative loadings influence the 24 h timepoint. According to PC-DFA loadings for the first discriminant function, all of the significant features selected by the network analysis are related to peak concentrations at 24 h (immediately after stroke onset), with the exception of E18 (tetracosapentaenoyl carnitine) which relates to the 13–15 days timepoint.

Of the features identified in the network analysis, 549.3797 × 13.29 and 550.3917 × 13.60 had the most negative values on the first discriminant function, identified as the [M]^+^ and [M + 1]^+^ adducts of platelet activating factor (plotted alongside significant EIDs in Fig. 3). This is closely followed by 532.3787 × 13.83 and 258.1126 × 12.97, identified as the [M + Na]^+^ and [M]^+^ adducts of 1-Alkyl-2-lyso-sn-glycero-3-phosphocholine and sn-Glycero-3-phosphocholine respectively. These glycerophosphocholines may be intermediates in the synthesis and metabolism of platelet activating factor. The next most negative loadings on the first discriminant function are the acyl carnitines E258, E23 and E116, plotted in Fig. 3 and listed in Table S2, as well as feature 284.2695 × 12.11, identified as the [M^+^] adduct of octadecanoate (n-C18:0).

The most positive value for the first discriminant function was for feature 327.22 × 16.04, the [M]^+^ adduct of 12,13-epoxy-9-hydroperoxy-10E-octadecenoate, followed by 445.3346 × 13.32 and 432.3594 × 13.64, identified as the [M]^+^ adducts of 13'-carboxy-γ-tocopherol and 13'-hydroxy-γ-tocopherol respectively.

For the second discriminant function, the most negative loadings were associated with 4-hydroperoxy-2-nonenal and several features identified as fatty acids: 297.2453 × 13.74, 279.2343 × 14.5, 279.2346 × 13.73, 280.2378 × 13.73 and 282.2534 × 13.85 assigned to the [M + H]^+^ adducts of (13S)-Hydroxyoctadecadienoic acid and (9Z,12Z,15Z)-Octadecatrienoic acid and the [M]^+^ adducts of α-Linolenic acid and (9Z)-Octadecenoic acid. The most positive loadings were 370.238 × 13.82 identified as the [M + H^+^] adduct of prostaglandin G1 and 414.2736 × 13.6 and 413.2699 × 19.07 identified as the [M + 1]^+^ and [M]^+^ adducts 11'-carboxy-α-tocotrienol.

**Figure 4:**
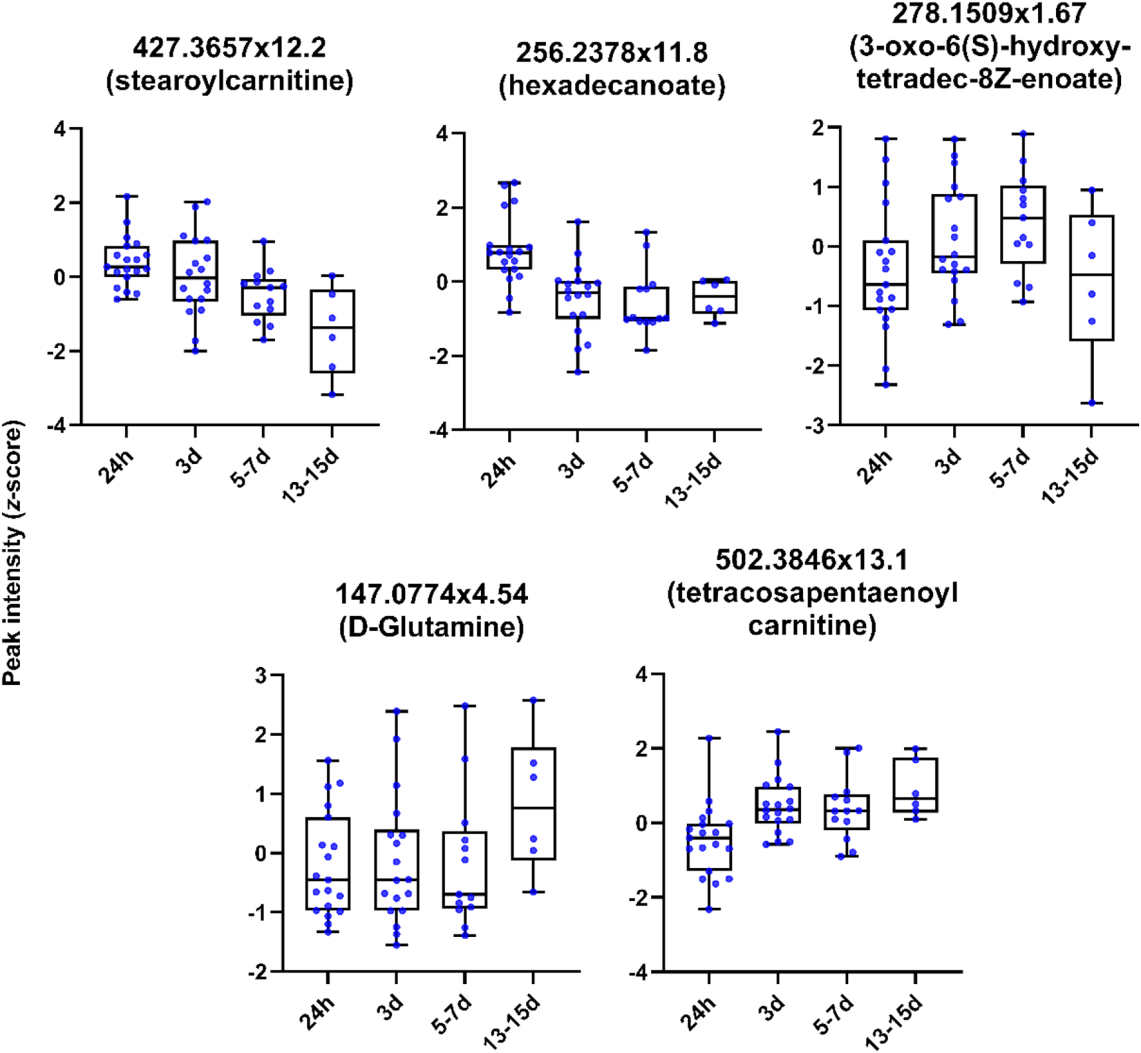
Boxplots (with median line, and whiskers of mininum and maximum value) for selected plasma metabolites showing change in intensity over time following stroke onset.

Figure 4 shows example box and whisker plots for metabolites selected from the loadings plot in Fig. 3. Features were selected to represent different pathways highlighted by molecular networking. There were no significant differences between metabolite levels when classified by stoke sub-type (posterior, total anterior and partial anterior circulation infarction), although sub-group sample sizes were small (Table 1). Future studies are therefore necessary in order to fully explore the impact that stroke vascular territory and mechanism may have on the circulating metabolome.

### Correlation between blood plasma metabolites, inflammatory mediators and breath metabolites

**Figure 5:**
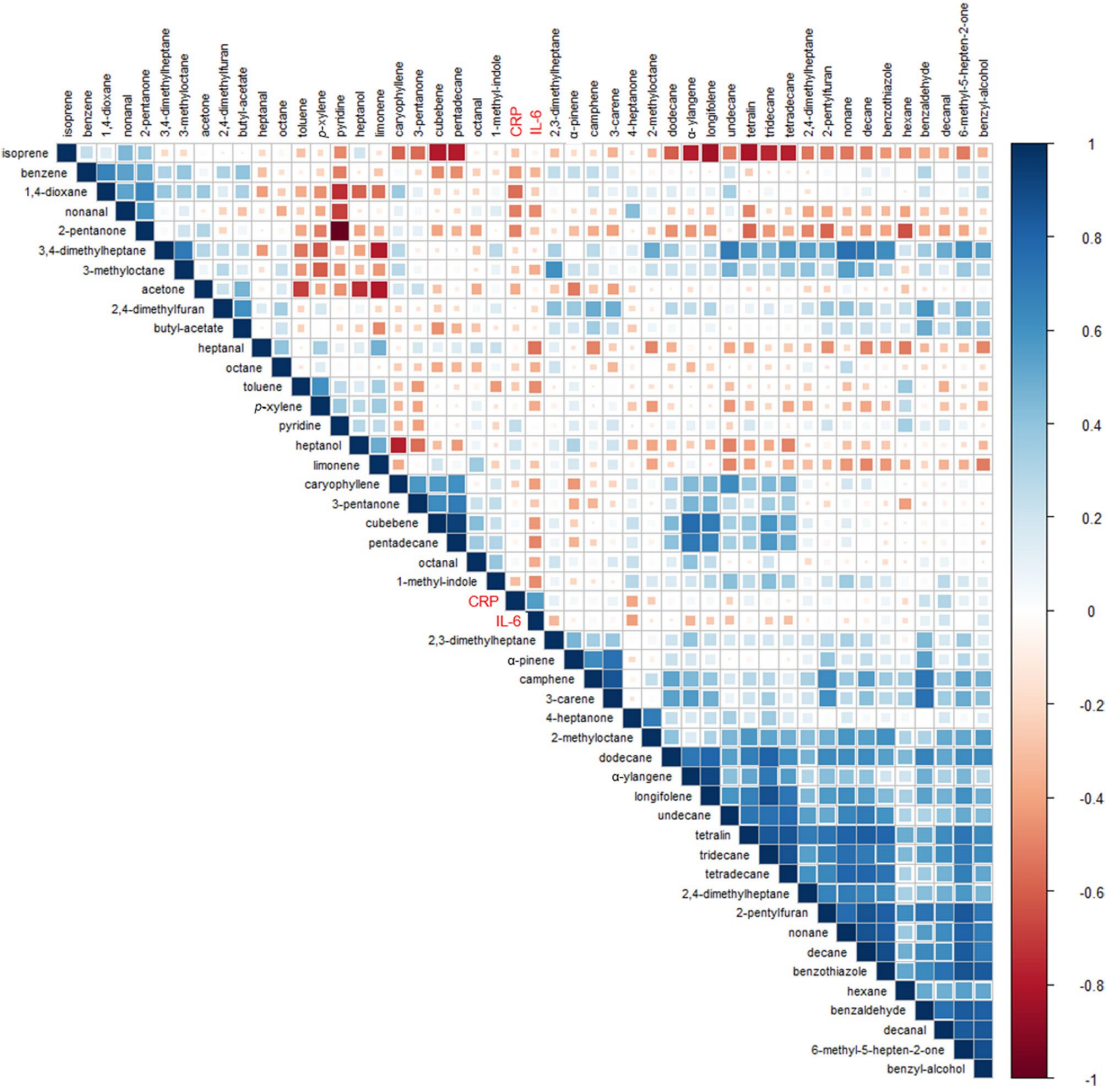
Correlations matrix plot of breath VOCs and inflammatory markers (CRP and IL-6) ordered by hierarchical clustering. Pearson’s correlation coefficients are shown by colour strength and circle size, where 1 is strong positive correlation, -1 is strong negative correlation, and 0 is no correlation.

To generate the two dimensional correlation plot presented in Fig. 5, VOCs were analysed for correlations with each other and with contemporaneously sampled CRP and IL-6 concentrations (*n* = 37). It is evident upon examination of Fig. 5 that VOCs of the same class (e.g. hydrocarbons) in many cases appear to correlate, in contrast to isoprene which showed a negative correlation with most hydrocarbons. The most negative isoprene correlation was with longifolene (*r* = − 0.65, *p* < 0.001). The top three positive correlations with CRP were for decanal (*r* = 0.38, *p* = 0.024), benzaldehyde (*r* = 0.34, *p* = 0.039) and pyridine (*r* = 0.28, *p* = 0.093). The top three negative correlations with CRP were observed for 1,4-dioxane (*r* = − 0.50, *p* = 0.002), 2-pentanone (*r* = − 0.40, *p* = 0.014) and hexane (*r* = − 0.29, *p* = 0.082). With regard to IL-6, the strongest positive correlation was with decanal (*r* = 0.45, *p* = 0.005), followed by benzyl alcohol (*r* = 0.25, *p* = 0.136) and tetralin (*r* = 0.19, *p* = 0.260), whereas the top three negatively correlated VOCs were nonane (*r* = − 0.40, *p* = 0.013), heptanal (*r* = − 0.40, *p* = 0.013) and nonanal (*r* = − 0.40, *p* = 0.014).

Of the 2420 MS features obtained following data processing of the blood plasma metabolites, 36 were found to significantly correlate with CRP and 53 with IL-6 (*p* value < 0.05) in contemporaneously acquired samples (*n* = 37), as shown in supplementary tables (Table S4 and S5). Of the features listed in Table S4, only 3 were identified in the network analysis: 200.1047 × 1.24, 470.3196 × 12.33 and 327.2313 × 13.17 which were tentatively assigned to Glutamyl-β-aminopropiononitrile, eicosatetranoyl carnitine and arachidonic acid, respectively. The acyl carnitine was negatively correlated with CRP (r = − 0.38, *p* = 0.038), whilst the other two compounds were positively correlated r = 0.45 (*p* = 0.001) and 0.38 (*p* = 0.041), respectively). Eicosatetranoyl carnitine is also listed in Table S5 and significantly correlates with IL-6 (r = − 0.39, *p* = 0.03) along with 3 other features: 442.2903 × 12.83, 507.3693 × 13.35, 363.2789 × 15.86, assigned to stearidonyl carnitine, Lysophosphatidylcholine and 12-hydroxyarachidonylethanolamide (with r = 0.50 (*p* = 0.002), − 0.39 (*p* = 0.038) and 0.37 (*p* = 0.050, respectively).

The top 3 correlations between peak levels of metabolites identified by molecular networking and stroke severity (NIHSS score) corresponded to the features: 307.1909 × 11.33 (*r*(20) = 0.51, *p* = 0.022), 550.3917 × 13.6 (*r*(20) = 0.51, *p* = 0.022) and 190.0121 × 1.41 (*r*(20) = 0.49, *p* = 0.028). These were assigned to the [M + H]^+^ adduct of 3-oxo-10-hydroxy-octadeca-6E,8E,12Z-trienoate, the [M]^+^ adduct of platelet activating factor and the [M + Na]^+^ adduct of quinolinate. The features that correlated most strongly with A^2^DS^2^ score include those assigned to 3-oxo-10-hydroxy-octadeca-6E,8E,12Z-trienoate and quinolinate (*r*(20) = 0.68, *p* < 0.001 and *r*(20) = 0.62, *p* = 0.003, respectively) and tetradecanoyl carnitine (*r*(20) = 0.62, *p* = 0.007).

Correlations between plasma metabolite features and breath VOCs were also assessed. Features with significant correlations (*p* < 0.05) with at least one breath VOC were extracted. A table of Pearson’s correlation coefficients from 816 blood plasma features with 46 breath VOCs is shown in supplementary file S6. VOC functional groups highly correlate with plasma features, for example with alkanes and terpenes (plasma feature retention time range of feature 757.6288 × 16.93, 7.24–7.65, and 17.86–19.61 min). These data suggest some blood plasma metabolites are directly linked to VOCs exhaled in breath. The features most correlated with decanal are 165.1651 × 15.00 (*r* = − 0.682, *p* < 0.001), 435.3097 × 19.58 (*r* = 0.595, *p* < 0.001). Pearson correlation coefficients for the significant empirical identities generated in the network analysis are shown in Table 3 for CRP, IL-6, decanal, and α-pinene.

## Discussion

Recent studies have demonstrated the potential that breath biomarkers have in monitoring inflammatory diseases^39^. However, establishing the endogenous metabolic relationship of many volatile breath components is still far from complete^40^ and challenges remain in standardising sampling and analysis^41^. In this study we investigated exhaled VOC and plasma metabolite changes in patients over 15 days after stroke onset. To the best of our knowledge, this is the first study to perform breath analysis of volatiles in stroke patients. We have demonstrated that recruitment is feasible, and sampling well-tolerated, in dysphagic patients and in patients with moderate or severe stroke, supporting further breath studies in acute stroke.

Exhaled decanal levels steadily increased over the first 7 days after stroke onset, in parallel to blood CRP and IL-6 concentration, suggesting that decanal is likely associated with systemic inflammation. Decanal was identified to MSI level 2 based on fragmentation pattern and retention index and targeted based on exact mass fragment. Although the most notable aldehyde marker of oxidative stress resulting from lipid peroxidation is malondialdehyde^42^, short chain aldehydes have been identified in the breath of individuals with chronic inflammatory diseases such as asthma^43^. Exhaled breath concentrations of decanal and other short chain aldehydes were also reported to increase in patients with oesophageal and gastric adenocarcinoma^44^. No other volatiles targeted in this study (including similar short chain aldehydes) mirrored inflammation markers in showing significant differences between the 24 h and 5–7 days timepoints.

The only other VOC that exhibited significant differences over the time course of this study was α-pinene, which decreased steadily across timepoints 24 h to 13–15 days and correlated well with other monoterpenes (e.g., camphene and 3-carene). As well as being detected in headspace during in vitro studies of several pathogenic species (e.g., *Aspergillus fumigatus*^45^) and in studies that identified exhaled markers of infectious diseases^16^, monoterpenes are ubiquitous in the ambient environment and are found in many food items. Changes in lifestyle following hospital admission are likely to be reflected in the volatile profile of patients’ breath, and decreasing exhaled α-pinene concentrations could be an example of this. Little is known on how changes to environment and diet may affect the breath of patients during medium and long-term hospital stay. Furthermore, washout of VOCs from the body is known to be compound dependent and complex^46,47^ and as studies have observed the storage of lipophilic compounds in the body^48^, changes to the exposome may manifest as gradual changes in exhaled breath composition.

Supervised multivariate analysis illustrated that plasma metabolite profiles could be classified by post-stroke time course. Annotation of plasma metabolite features with their putative pathways and identities were predicted from metabolic network analysis. Five metabolites associated with the carnitine shuttle were putatively identified as tetradecanoyl carnitine, stearoylcarnitine, tetracosapentaenoyl carnitine, tetracosatetraenoyl carnitine and L-palmitoylcarnitine, with the latter additionally involved in saturated fatty acid β-oxidation. Carnitine transports fatty acids from stores into mitochondria for energy production, and have been found to be down regulated in frailty in the elderly^49^. Studies have shown elevated serum acyl carnitine in stroke patients on admission compared to healthy controls^50,51^. Our results are consistent with an increase in energy requirements symptomatic of ischemia followed by gradually depleted concentrations after stroke. However, one acyl carnitine, tetracosapentaenoyl carnitine continued to increase throughout the duration of the sampling period. This conflicting behaviour is also reflected in the correlation coefficients calculated for acyl carnitines and IL-6, e.g., eicosatetranoyl negatively correlated whilst stearidonyl carnitine positively correlated with IL-6. In general, the time profiles for different acyl carnitines exhibit a diverse range, perhaps reflecting the numerous roles that acyl carnitines play in stroke recovery for energy production and neuroprotection.

Peak levels of platelet activating factor identified in the network analysis were found to weakly correlate with stroke severity. The initial peak in levels of platelet activating factor and strong downward trend that followed was mirrored by several acyl carnitines and octadecanoate (n-C18:0). Another short chain fatty acid, hexadecanoate (n-C16:0), is located near L-palmitoylcarnitine on the PC-DFA loadings plot, supporting their feature assignment and grouping by biochemical pathway. Platelet activating factor is known to be a potent mediator of inflammation and is synthesised in the injured brain^52^.

Polyunsaturated fatty acids (PUFA) identified as 8(*R*)-hydroxy-hexadeca-2E,6E,10Z-trienoate (E55) and 6(*R*)-hydroxy-tetradeca-2E,4E,8Z-trienoate (E9), are associated with 3-oxo-10*R*-octadecatrienoate β-oxidation. According to PC-DFA loadings, these PUFAs had a strong influence on the first discriminant function in the negative direction, and therefore were associated with 24 h timepoint (the first sample after stroke onset). Furthermore, these PUFAs were negatively correlated to inflammatory markers and breath decanal which showed increased concentrations post-stroke, indicating a relationship between inflammatory markers, blood metabolites, and breath volatiles. A study by Jiang et al*.*^53^ identified similar PUFAs that were predictive of stroke and found that concentrations were increased in serum compared to age and gender-matched controls. However, decreased levels of several fatty acids have also been associated with increased stroke risk. It is interesting to note that the other PUFAs identified in the network analysis peak at progressively later times according to their loadings, as depicted in Fig. 3.

Quinolinate is involved in tryptophan metabolism and is thought to be a neurotoxic metabolite produced in the kynurenine pathway originating from the catabolism of tryptophan by indolamine 2,3-dioxygenase, involved in inducing oxidative stress and activating NMDA receptor activity^54,55^. Brouns et al*.* analysed TRP and its metabolites in stroke patients and found that 3-hydroxyanthranilate (and quinolinate as a direct precursor of 3-hydroxyanthranilate) significantly declined in concentration from admission onwards^55^. They concluded tryptophan degradation, which also correlated with CRP, was predictive of acute stroke severity and long-term stroke outcome. This is consistent with our study which shows where quinolinate was found to correlate with NIHSS score.

Purinergic signalling is involved in ischemia and stroke^56^ and stroke severity has been linked with elevation in blood purines (adenosine, inosine and xanthine)^57^. However, as yet another example of metabolic duality, decreased adenosine levels have also been reported^53^ and both elevated and decreased glutamine levels have been previously observed in ischemic stroke^51,58,59^. This is also reflected in the present study whereby feature 176.0315 × 1.37 (attributed to monodehydroascorbate and glucurono-6,3-lactone) had negative loadings along the second discriminant function and peak levels were observed for the 24 h timepoint, whilst loadings for all other purine metabolites (guanine, inosine, urate, xanthine, adenosine and 5-Phospho-β-D-ribosylamine) were positive indicating that they peak at progressively later times. This biochemistry is complex, for example, monodehydroascorbate and glucurono-6,3-lactone are also involved in D4&E4-neuroprostanes formation and ascorbate and aldarate metabolism. Nevertheless, the conflicting results obtained in previous metabolomics studies may well reflect the metabolic dynamics of post-stroke inflammation and repair.

A range of VOCs were targeted in breath representing different chemical properties and potential pathways relating to their roles as metabolites. Through breath VOC intra-correlation analysis, we have shown that several exhaled compounds with similar structural chemistry correlate with one another, for example branched and straight chain alkanes or structural isomers of monoterpenes. Furthermore, several breath VOCs correlated well with both plasma CRP and with clusters of UHPLC features, supporting the view that certain volatiles are endogenously produced from metabolic processes.

Follow-on studies investigating stroke biomarkers or post-stroke complications such as stroke-associated pneumonia must consider increased sample sizes and appropriate non-stroke control comparisons. This can be achieved through multi-site or external validation studies. Improvements in analytical methodologies may also engender confidence in post-stroke prognostic biomarker development. Future studies may consider using a lipidomics approach as several lipid metabolites were identified. In addition, parallel methods with chromatography phases suitable for hydrophilic compounds (e.g. HILIC or normal phase) will be advantageous to resolving polar metabolites and therefore compound identification for example features in this study with retention times under 2 min (E96, E311). It should be noted that our entry criteria were relatively strict, requiring participants to be recruited within 24 h of stroke onset, and at high risk of pneumonia, thus limiting the number of eligible participants. Whilst our findings relating to metabolomics and inflammation should only be applied to this group, it is reasonable to assume that our main findings around feasibility could be extrapolated to those with less severe strokes and at later timepoints after onset.

Infections, particularly pneumonia, commonly complicate stroke and are associated with worse clinical outcomes. Currently, there is a need for accurate biomarkers to guide prediction and treatment of stroke-associated pneumonia and inform prognosis. Due to the small sample size and modest A^2^DS^2^ threshold, no patients developed CDC-confirmed stroke-associated pneumonia. Even in the absence of any confirmed pneumonia, our data provide preliminary insights into the relationships between peripheral inflammatory markers and metabolic pathways in acute stroke. The principles underlying our breath sampler have been further developed through the Breathe-Free consortium^60^, and a commercial sampler based on this concept is now available, further facilitating larger scale clinical studies^27,61^. Limited strategies are available to effectively prevent pneumonia in acute stroke, and antibiotic treatment when pneumonia is suspected remains the cornerstone of clinical management. Predicting those at risk of pneumonia complicating stroke is critical in directing monitoring and potential preventive therapies as well as resource allocation.

## Conclusion

We found changes in concentrations of endogenous metabolites over time which may be indicative of stroke-associated inflammatory activity. We observed multiple positive and negative correlations across exhaled breath components and blood plasma metabolites which demonstrate a dynamic response, potentially a consequence of intrinsic neurotoxic and neuroprotective mechanisms. Metabolites from blood plasma and breath samples correlated with the established inflammatory markers CRP and IL-6. These results illustrate the complexity of the endogenous inflammatory cascade during evolution of acute and subacute stroke and into the recovery phase, and warrant future investigation towards potential clinical application.

Our study shows serial breath sampling is feasible and well-tolerated in patients with acute stroke. We have also explored relationships between plasma inflammatory markers, plasma metabolites, and breath volatiles which motivates additional multi-omics and multi-compartment analyses to confirm metabolic origins. Follow on studies are required to validate our results and to further explore the breath volatilome for novel biomarkers, particularly for predicting or diagnosing stroke-associated pneumonia and for prognostication.

## Supplementary Information

Supplementary Information 1.

Supplementary Information 2.
