# Supplementary material for: Breath and plasma metabolomics to assess inflammation in acute stroke
Source: Sci Rep. 2021 Nov 9;11:21949. doi: 10.1038/s41598-021-01268-5 (PMC8578671; doi:10.1038/s41598-021-01268-5)
Supplement: Supplementary file 1 — Supplementary Information 1. [file 41598_2021_1268_MOESM1_ESM.docx]

**Breath and plasma metabolomics to assess inflammation in acute stroke**

**SUPPLEMENTARY MATERIAL**

**Table S1. VOCs targeted during data pre-processing**

| # | **Compound** | **EI fragment** | **Target ion**  ***m*/*z*** | **RT / [min]** | **ICC (95% CI)** |
| --- | --- | --- | --- | --- | --- |
| *1* | isoprene^(1)^ | [C_5_H_7_]^+^ | 67.0548 | 2.00 | 0.79 (0.25-0.97) |
| *2* | acetone^(1)^ | [C_3_H_6_O]^+^ | 58.0419 | 2.05 | 0.78 (0.19-0.97) |
| *3* | *n-*hexane^(2)^ | [C_6_H_14_]^+^ | 86.1096 | 2.22 | < 0 |
| *4* | benzene^(1)^ | [C_6_H_6_]^+^ | 78.0470 | 2.71 | 0.92 (0.69-0.99) |
| *5* | 2-pentanone^(2)^ | [C_5_H_10_O]^+^ | 86.0732 | 2.90 | 0.79 (0.2-0.98) |
| *6* | 3-pentanone^(1)^ | [C_3_H_5_O]^+^ | 57.0340 | 3.00 | 0.81 (0.2-0.98) |
| *7* | 1,4-dioxane^(1)^ | [C_4_H_8_O_2_]^+^ | 88.0524 | 3.14 | 0.94 (0.75-0.99) |
| *8* | 2,4-dimethylfuran^(2)^ | [C_6_H_8_O]^+^ | 96.0575 | 3.20 | 0.35 (-1.46-0.93) |
| *9* | pyridine^(1)^ | [C_5_H_5_N]^+^ | 79.0422 | 3.57 | 0.24 (-1.75-0.91) |
| *10* | toluene^(1)^ | [C_7_H_7_]^+^ | 91.0548 | 3.93 | 0.84 (0.38-0.98) |
| *11* | *n-*octane^(2)^ | [C_5_H_11_]^+^ | 71.0861 | 4.38 | 0.8 (0.19-0.98) |
| *12* | butyl acetate^(1)^ | [C_3_H_5_O_2_]^+^ | 73.0290 | 4.62 | 0.75 (-0.1-0.97) |
| *13* | 2,4-dimethylheptane^(2)^ | [C_9_H_2_0]^+^ | 128.1565 | 4.77 | 0.94 (0.77-0.99) |
| *14* | 2,3-dimethylheptane^(2)^ | [C_6_H_13_]^+^ | 85.1017 | 5.45 | 0.78 (0.22-0.97) |
| *15* | 3,4-dimethylheptane^(2)^ | [C_9_H_2_0]^+^ | 128.1565 | 5.59 | 0.91 (0.62-0.99) |
| *16* | 4-heptanone^(2)^ | [C_7_H_14_O]^+^ | 114.1045 | 5.70 | 0.67 (-0.12-0.96) |
| *17* | 2-methyloctane^(2)^ | [C_8_H_17_]^+^ | 113.1330 | 5.71 | 0.52 (-0.61-0.94) |
| *18* | 3-methyloctane^(2)^ | [C_6_H_13_]^+^ | 85.1017 | 5.79 | 0.87 (0.53-0.99) |
| *19* | *p-*xylene^(1)^ | [C_7_H_7_]^+^ | 91.0548 | 5.79 | 0.35 (-2.16-0.93) |
| *20* | *n-*nonane^(1)^ | [C_9_H_2_0]^+^ | 128.1565 | 6.41 | 0.77 (0.15-0.97) |
| *21* | heptanal^(2)^ | [C_7_H_12_]^+^ | 96.0939 | 6.45 | 0.78 (0.13-0.97) |
| *22* | α-pinene^(2)^ | [C_7_H_9_]^+^ | 93.0704 | 7.24 | 0.09 (-1.1-0.87) |
| *23* | camphene^(2)^ | [C_7_H_9_]^+^ | 93.0704 | 7.66 | 0.44 (-2.27-0.94) |
| *24* | benzaldehyde^(1)^ | [C_7_H_5_O]^+^ | 105.0340 | 7.88 | 0.97 (0.87-1) |
| *25* | 1-heptanol^(1)^ | [C_5_H_10_]^+^ | 70.0783 | 8.13 | 0.96 (0.86-1) |
| *26* | 6-Methyl-5-hepten-2-one^(2)^ | [C_8_H_14_O]^+^ | 126.1045 | 8.45 | 0.6 (-0.23-0.95) |
| *27* | 2-pentylfuran^(2)^ | [C_9_H_14_O]^+^ | 138.1045 | 8.61 | < 0 |
| *28* | *n-*decane^(1)^ | [C_10_H_22_]^+^ | 142.1722 | 8.80 | 0.8 (0.2-0.98) |
| *29* | octanal^(2)^ | [C_8_H_16_O]^+^ | 128.1201 | 8.89 | 0.97 (0.89-1) |
| *30* | 3-carene^(1)^ | [C_7_H_9_]^+^ | 93.0704 | 9.09 | 0.06 (-1.77-0.88) |
| *31* | limonene^(1)^ | [C_5_H_8_]^+^ | 68.0626 | 9.61 | < 0 |
| *32* | benzyl alcohol^(2)^ | [C_7_H_8_O]^+^ | 108.0575 | 9.72 | 0.78 (0.15-0.98) |
| *33* | *n-*undecane^(1)^ | [C_11_H_24_]^+^ | 156.1878 | 11.34 | 0.98 (0.93-1) |
| *34* | nonanal^(1)^ | [C_7_H_14_]^+^ | 98.1096 | 11.47 | 0.87 (0.44-0.99) |
| *35* | tetralin^(2)^ | [C_10_H_12_]^+^ | 132.0939 | 12.92 | 0.98 (0.9-1) |
| *36* | *n-*dodecane^(1)^ | [C_12_H_26_]^+^ | 170.2035 | 13.80 | 0.97 (0.88-1) |
| *37* | decanal^(2)^ | [C_10_H_20_O]^+^ | 156.1514 | 13.96 | 0.77 (0.2-0.97) |
| *38* | benzothiazole^(2)^ | [C_7_H_5_NS]^+^ | 135.0143 | 14.55 | 0.88 (0.49-0.99) |
| *39* | 1-methylindole^(1)^ | [C_9_H_9_N]^+^ | 131.0735 | 15.40 | 0.55 (-1.04-0.95) |
| *40* | *n-*tridecane^(1)^ | [C_13_H_28_]^+^ | 184.2191 | 16.15 | 0.83 (0.28-0.98) |
| *41* | cubebene^(2)^ | [C_15_H_24_]^+^ | 204.1878 | 18.01 | 0.83 (0.37-0.98) |
| *42* | α-ylangene^(2)^ | [C_12_H_17_]^+^ | 161.1330 | 18.22 | 0.76 (0.13-0.97) |
| *43* | *n-*tetradecane^(1)^ | [C_14_H_30_]^+^ | 198.2348 | 18.42 | 0.77 (0.12-0.97) |
| *44* | longifolene^(2)^ | [C_12_H_17_]^+^ | 161.1330 | 18.43 | 0.69 (-0.04-0.96) |
| *45* | caryophyllene^(2)^ | [C_15_H_24_]^+^ | 204.1880 | 18.78 | 0.95 (0.8-0.99) |
| *46* | *n-*pentadecane^(1)^ | [C_15_H_32_]^+^ | 212.2504 | 20.45 | 0.38 (-1.36-0.93) |

RT = retention time; ICC = Intraclass correlation coefficient; (1) identified to MSI level 1; (2) identified to MSI level 2

**Table S2. Statistical test results (Kruskal-Wallis test) from timepoint group comparisons of all breath VOCs**

| **Compound** | **KW**  **statistic** | ***p*-value** | **No.**  **values** |  |  | **Compound** | **KW**  **statistic** | ***p*-value** | **No.**  **values** |
| --- | --- | --- | --- | --- | --- | --- | --- | --- | --- |
| α-pinene | 9.6220 | 0.0221 | 45 |  |  | benzaldehyde | 1.3640 | 0.7140 | 46 |
| decanal | 7.5110 | 0.0573 | 37 |  |  | *n-*pentadecane | 1.3330 | 0.7214 | 46 |
| 3-carene | 5.7910 | 0.1222 | 46 |  |  | limonene | 1.2290 | 0.7460 | 47 |
| sulcatone | 5.4200 | 0.1435 | 47 |  |  | octanal | 1.2030 | 0.7522 | 37 |
| 2-pentanone | 4.2290 | 0.2378 | 44 |  |  | benzyl alcohol | 1.1800 | 0.7577 | 47 |
| α-ylangene | 4.1900 | 0.2417 | 45 |  |  | 2,4-dimethylheptane | 1.0820 | 0.7814 | 40 |
| tetralin | 4.1240 | 0.2484 | 44 |  |  | cubebene | 1.0620 | 0.7864 | 45 |
| benzothiazole | 3.5610 | 0.3129 | 47 |  |  | 2,4-dimethylfuran | 1.0590 | 0.7869 | 45 |
| toluene | 3.3100 | 0.3463 | 47 |  |  | *p*-xylene | 1.0380 | 0.7919 | 47 |
| nonanal | 3.0560 | 0.3830 | 46 |  |  | 3,4-dimethylheptane | 0.9328 | 0.8175 | 44 |
| acetone | 2.8650 | 0.4128 | 46 |  |  | 2-pentylfuran | 0.8484 | 0.8379 | 45 |
| camphene | 2.7980 | 0.4238 | 45 |  |  | *n-*tridecane | 0.8030 | 0.8487 | 45 |
| 3-pentanone | 2.1270 | 0.5464 | 38 |  |  | 4-heptanone | 0.7752 | 0.8554 | 45 |
| *n-*tetradecane | 1.8980 | 0.5938 | 45 |  |  | benzene | 0.6005 | 0.8963 | 47 |
| 1,4-dioxane | 1.8550 | 0.6030 | 46 |  |  | *n-*octane | 0.5799 | 0.9010 | 47 |
| pyridine | 1.8140 | 0.6120 | 47 |  |  | *n-*hexane | 0.5229 | 0.9138 | 45 |
| 1-methylindole | 1.5930 | 0.6609 | 38 |  |  | butyl acetate | 0.5199 | 0.9145 | 47 |
| 2-methyloctane | 1.5900 | 0.6617 | 44 |  |  | 1-heptanol | 0.4144 | 0.9373 | 34 |
| heptanal | 1.5370 | 0.6738 | 47 |  |  | *n-*nonane | 0.4039 | 0.9394 | 44 |
| *n-*decane | 1.4780 | 0.6874 | 46 |  |  | *n-*undecane | 0.2692 | 0.9657 | 39 |
| *n-*dodecane | 1.3980 | 0.7059 | 46 |  |  | 2,3-dimethylheptane | 0.2594 | 0.9675 | 42 |
| caryophyllene | 1.3790 | 0.7104 | 41 |  |  | 3-methyloctane | 0.1171 | 0.9897 | 41 |
| longifolene | 1.3700 | 0.7127 | 46 |  |  | benzaldehyde | 1.3640 | 0.7140 | 46 |

KW= Kruskal-Wallis

**Table S3. List of all features identified from molecular networking analysis for key pathways**

| **Feature** | **Predicted metabolite identification (MSI level 2)** | | | **PC-DFA** | |
| --- | --- | --- | --- | --- | --- |
| ID (*m*/*z* x RT/[min]) | **Empirical ID** | **Compound Name** | **Adduct** | **DF1** | **DF2** |
| ***Carnitine shuttle*** | |  |  |  |  |
| 526.3817x13.49 | E258^*^ | tetracosatetraenoyl carnitine | [M+Na]^+^ | -0.035 | -0.028 |
| 372.3142x11.50 | E83^*^ | tetradecanoyl carnitine | [M+H]^+^ | -0.034 | -0.006 |
| 470.3196x12.33 | E116 | eicosatetranoyl carnitine | [M+Na]^+^ | -0.031 | -0.031 |
| 496.335x12.52 | E33 | clupanodonyl carnitine | [M+Na]^+^ | -0.030 | -0.032 |
| 399.3339x11.69 | E286^*^ | palmitoylcarnitine | [M]^+^ | -0.024 | -0.006 |
| 444.3081x12.61 | E23 | α-linolenyl carnitine | [M+Na]^+^ | -0.023 | -0.007 |
| 371.3022x11.10 | E165^*^ | tetradecanoyl carnitine | [M]^+^ | -0.021 | -0.004 |
| 427.3657x12.20 | E68^*^ | stearoylcarnitine | [M]^+^ | -0.020 | -0.011 |
| 413.3529x11.86 | E24 | heptadecanoyl carnitine | [M]^+^ | -0.017 | -0.028 |
| 425.35x11.88 | E229 | elaidic carnitine | [M]^+^ | -0.016 | -0.006 |
| 419.3047x12.80 | E79 | stearidonyl carnitine | [M]^+^ | -0.009 | 0.008 |
| 450.3622x12.08 | E77 | dihomo-γ-linolenyl carnitine | [M+H]^+^ | -0.006 | -0.034 |
| 218.1405x1.46 | E17 | propionyl-carnitine | [M+H]^+^ | 0.015 | -0.013 |
| 539.4929 x13.33 | E343 | hexacosanoyl carnitine | [M+H]^+^ | -0.016 | -0.028 |
| 502.3846x13.10 | E18^*^ | tetracosapentaenoyl carnitine | [M]^+^ | 0.030 | 0.033 |
| ***Saturated fatty acids β-oxidation*** | | |  |  |  |
| 256.2378x11.8 | E49^*^ | Hexadecanoate (n-C16:0) | [M]^+^ | -0.0261 | -0.0172 |
| 399.3339x11.69 | E286^*^ | palmitoylcarnitine | [M]^+^ | -0.0244 | -0.0061 |
| ***3-oxo-10R-octadecatrienoate β-oxidation*** | | |  |  |  |
| 213.1503x9.81 | E189* | 6-hydroxy-tetradeca-2E,4E,8Z-trienoate | [M]^+^ | -0.0192 | -0.0041 |
| 237.1482x10.78 | E304^*^ | 8-hydroxy-hexadeca-2E,6E,10Z-trienoate | [M]^+^ | -0.0228 | -0.0062 |
| 265.1801x11.76 | E97 | 3,6-dihydroxy-tetradec-8Z-enoate | [M+Na]^+^ | 0.0128 | 0.0220 |
| 280.1651x11.41 | E350 | 3-oxo-10-hydroxy-octadeca-6E,8E,12Z-trienoate | [M]^+^ | -0.0139 | 0.0117 |
| 307.1909x11.33 | E241 | 3-oxo-6-hydroxy-tetradec-8Z-enoate | [M+Na]^+^ | 0.0151 | 0.0207 |
| 278.1509x1.67 | E285 | 3-oxo-8-hydroxy-hexadeca-6E,10Z-dienoate | [M]^+^ | -0.0153 | 0.0019 |
| 281.1756x10.86 | E97 | 3,6-dihydroxy-tetradec-8Z-enoate | [M+Na]^+^ | 0.0128 | 0.0220 |
| 280.1651x11.41 | E350 | 3-oxo-10-hydroxy-octadeca-6E,8E,12Z-trienoate | [M]^+^ | -0.0139 | 0.0117 |
| 307.1909x11.33 | E241 | 3-oxo-6-hydroxy-tetradec-8Z-enoate | [M+Na]^+^ | 0.0151 | 0.0207 |
| 278.1509x1.67 | E285 | 3-oxo-8-hydroxy-hexadeca-6E,10Z-dienoate | [M]^+^ | -0.0153 | 0.0019 |
| 281.1756x10.86 | E304 | 8-hydroxy-hexadeca-2E,6E,10Z-trienoate | [M]^+^ | -0.0228 | -0.0062 |
| 265.1801x11.76 | E122 | 4-hydroxy-dodec-6Z-enoate | [M]^+^ | -0.0190 | 0.0098 |
| ***Purine metabolism*** | | |  |  |  |
| 176.0315x1.37 | E96^*^ | monodehydroascorbate | [M+H]^+^] | -0.020 | -0.007 |
| 176.0315x1.37 | E311^*^ | glucurono-6,3-lactone | [M]^+^ | -0.020 | -0.007 |
| 291.0729x1.37 | E156 | Inosine | [M+Na]^+^ | -0.014 | 0.001 |
| 230.0408x6.45 | E269 | 5-Phospho-β-D-ribosylamine | [M+H]^+^ | -0.020 | 0.003 |
| 152.033x1.14 | E178 | Xanthine | [M]^+^ | -0.006 | 0.003 |
| 267.099x9.31 | E253 | Adenosine | [M]^+^ | -0.003 | 0.005 |
| 152.0553x1.09 | E124 | Guanine | [M+H]^+^ | 0.005 | 0.011 |
| 169.0366x1.01 | E108 | Urate | [M+H]^+^ | -0.002 | 0.013 |
| 147.0774x4.54 | E62 | Glutamine | [M+H]^+^ | 0.002 | 0.023 |

* Significant EIDs

**Table S4. A list of plasma metabolite features which significantly correlated with CRP concentration ordered by most significant**

| **Feature** | ***m/z*** | **Retention Time (min)** | **Pearson's *r*** | **FDR corrected *p*-value** |
| --- | --- | --- | --- | --- |
| 717.5600x15.06 | 717.5600 | 15.06 | 0.52 | 0.0012 |
| 437.3350x12.84 | 437.3350 | 12.84 | 0.46 | 0.0075 |
| 993.6885x12.96 | 993.6885 | 12.96 | -0.45 | 0.0093 |
| 200.1047x1.24 | 200.1047 | 1.24 | 0.45 | 0.0099 |
| 462.3472x12.94 | 462.3472 | 12.94 | 0.45 | 0.0100 |
| 991.6820x12.97 | 991.6820 | 12.97 | -0.44 | 0.0122 |
| 491.2989x12.33 | 491.2989 | 12.33 | -0.44 | 0.0130 |
| 719.5758x15.59 | 719.5758 | 15.59 | 0.43 | 0.0157 |
| 263.1409x12.33 | 263.1409 | 12.33 | -0.42 | 0.0183 |
| 275.2798x10.38 | 275.2798 | 10.38 | -0.42 | 0.0201 |
| 466.3338x13.46 | 466.3338 | 13.46 | -0.41 | 0.0215 |
| 992.6853x12.97 | 992.6853 | 12.97 | -0.41 | 0.0223 |
| 302.1472x11.42 | 302.1472 | 11.42 | 0.41 | 0.0236 |
| 503.2576x12.82 | 503.2576 | 12.82 | 0.40 | 0.0279 |
| 132.1037x1.54 | 132.1037 | 1.54 | 0.40 | 0.0283 |
| 253.6341x12.33 | 253.6341 | 12.33 | -0.40 | 0.0284 |
| 262.6393x12.35 | 262.6393 | 12.35 | -0.40 | 0.0294 |
| 399.3124x12.84 | 399.3124 | 12.84 | 0.40 | 0.0308 |
| 664.4629x15.73 | 664.4629 | 15.73 | 0.39 | 0.0325 |
| 254.1359x12.33 | 254.1359 | 12.33 | -0.39 | 0.0339 |
| 450.3017x15.46 | 450.3017 | 15.46 | -0.39 | 0.0341 |
| 155.0116x15.00 | 155.0116 | 15.00 | -0.39 | 0.0341 |
| 830.5771x15.54 | 830.5771 | 15.54 | -0.39 | 0.0364 |
| 468.3132x12.34 | 468.3132 | 12.34 | -0.39 | 0.0371 |
| 470.3196x12.33 | 470.3196 | 12.33 | -0.38 | 0.0383 |
| 538.5235x15.57 | 538.5235 | 15.57 | 0.38 | 0.0401 |
| 327.2313x13.17 | 327.2313 | 13.17 | 0.38 | 0.0408 |
| 481.3531x13.22 | 481.3531 | 13.22 | -0.38 | 0.0409 |
| 429.3811x12.48 | 429.3811 | 12.48 | -0.38 | 0.0412 |
| 292.6577x12.94 | 292.6577 | 12.94 | 0.38 | 0.0416 |
| 480.3496x13.22 | 480.3496 | 13.22 | -0.38 | 0.0419 |
| 552.4070x14.00 | 552.4070 | 14.00 | -0.38 | 0.0447 |
| 410.3022x12.97 | 410.3022 | 12.97 | 0.38 | 0.0454 |
| 438.3830x12.84 | 438.3830 | 12.84 | -0.37 | 0.0467 |
| 102.9711x2.11 | 102.9711 | 2.11 | -0.37 | 0.0496 |
| 185.0783x12.87 | 185.0783 | 12.87 | -0.37 | 0.0497 |

**Table S5. A list of plasma metabolite features which significantly correlated with IL-6 concentration ordered by most significant**

| **Feature** | ***m/z*** | **Retention Time (min)** | **Pearson's *r*** | **FDR corrected *p*-value** |
| --- | --- | --- | --- | --- |
| 503.2576x12.82 | 503.2576 | 12.82 | 0.58 | 0.0001 |
| 462.3472x12.94 | 462.3472 | 12.94 | 0.58 | 0.0001 |
| 538.5235x15.57 | 538.5235 | 15.57 | 0.52 | 0.0012 |
| 441.2869x12.83 | 441.2869 | 12.83 | 0.51 | 0.0016 |
| 442.2903x12.83 | 442.2903 | 12.83 | 0.50 | 0.0023 |
| 436.3315x12.84 | 436.3315 | 12.84 | 0.49 | 0.0033 |
| 503.3013x12.56 | 503.3013 | 12.56 | -0.48 | 0.0043 |
| 502.2981x12.56 | 502.2981 | 12.56 | -0.47 | 0.0053 |
| 527.2583x12.59 | 527.2583 | 12.59 | 0.46 | 0.0062 |
| 437.3350x12.84 | 437.3350 | 12.84 | 0.46 | 0.0072 |
| 524.2800x12.55 | 524.2800 | 12.55 | -0.45 | 0.0092 |
| 271.3218x12.44 | 271.3218 | 12.44 | 0.45 | 0.0102 |
| 336.3294x12.67 | 336.3294 | 12.67 | 0.44 | 0.0108 |
| 264.2710x15.01 | 264.2710 | 15.01 | 0.44 | 0.0112 |
| 327.2048x12.85 | 327.2048 | 12.85 | 0.43 | 0.0139 |
| 491.2989x12.33 | 491.2989 | 12.33 | -0.42 | 0.0172 |
| 538.5249x15.01 | 538.5249 | 15.01 | 0.42 | 0.0181 |
| 507.8299x12.97 | 507.8299 | 12.97 | 0.42 | 0.0182 |
| 509.3366x12.60 | 509.3366 | 12.60 | 0.42 | 0.0193 |
| 476.7984x12.85 | 476.7984 | 12.85 | 0.41 | 0.0221 |
| 415.2884x12.85 | 415.2884 | 12.85 | 0.41 | 0.0226 |
| 488.7988x12.79 | 488.7988 | 12.79 | 0.41 | 0.0232 |
| 993.6885x12.96 | 993.6885 | 12.96 | -0.41 | 0.0232 |
| 500.2824x12.25 | 500.2824 | 12.25 | -0.41 | 0.0236 |
| 270.6265x12.53 | 270.6265 | 12.53 | -0.41 | 0.0237 |
| 992.6853x12.97 | 992.6853 | 12.97 | -0.41 | 0.0249 |
| 511.2804x12.58 | 511.2804 | 12.58 | 0.40 | 0.0270 |
| 352.3250x12.65 | 352.3250 | 12.65 | 0.40 | 0.0276 |
| 210.1309x7.56 | 210.1309 | 7.56 | -0.40 | 0.0280 |
| 392.3473x11.90 | 392.3473 | 11.90 | -0.40 | 0.0282 |
| 664.4629x15.73 | 664.4629 | 15.73 | 0.40 | 0.0304 |
| 238.1262x7.56 | 238.1262 | 7.56 | -0.39 | 0.0335 |
| 262.6393x12.35 | 262.6393 | 12.35 | -0.39 | 0.0338 |
| 466.2906x12.60 | 466.2906 | 12.60 | 0.39 | 0.0343 |
| 470.3196x12.33 | 470.3196 | 12.33 | -0.39 | 0.0348 |
| 178.1245x8.94 | 178.1245 | 8.94 | -0.39 | 0.0352 |
| 468.3132x12.34 | 468.3132 | 12.34 | -0.39 | 0.0363 |
| 507.3693x13.35 | 507.3693 | 13.35 | -0.39 | 0.0380 |
| 991.6820x12.97 | 991.6820 | 12.97 | -0.38 | 0.0390 |
| 380.7559x15.85 | 380.7559 | 15.85 | 0.38 | 0.0395 |
| 325.0997x9.30 | 325.0997 | 9.30 | 0.38 | 0.0408 |
| 663.4593x15.73 | 663.4593 | 15.73 | 0.38 | 0.0410 |
| 408.3725x13.13 | 408.3725 | 13.13 | 0.38 | 0.0413 |
| 396.7497x15.38 | 396.7497 | 15.38 | -0.38 | 0.0423 |
| 754.5459x15.36 | 754.5459 | 15.36 | -0.38 | 0.0443 |
| 706.6004x16.18 | 706.6004 | 16.18 | 0.38 | 0.0455 |
| 719.5758x15.59 | 719.5758 | 15.59 | 0.37 | 0.0468 |
| 310.1519x1.06 | 310.1519 | 1.06 | 0.37 | 0.0482 |
| 419.3047x12.80 | 419.3047 | 12.80 | 0.37 | 0.0484 |
| 253.6341x12.33 | 253.6341 | 12.33 | -0.37 | 0.0484 |
| 482.4103x12.84 | 482.4103 | 12.84 | 0.37 | 0.0494 |
| 363.2789x15.86 | 363.2789 | 15.86 | 0.37 | 0.0498 |
| 283.1781x12.86 | 283.1781 | 12.86 | 0.37 | 0.0499 |

**
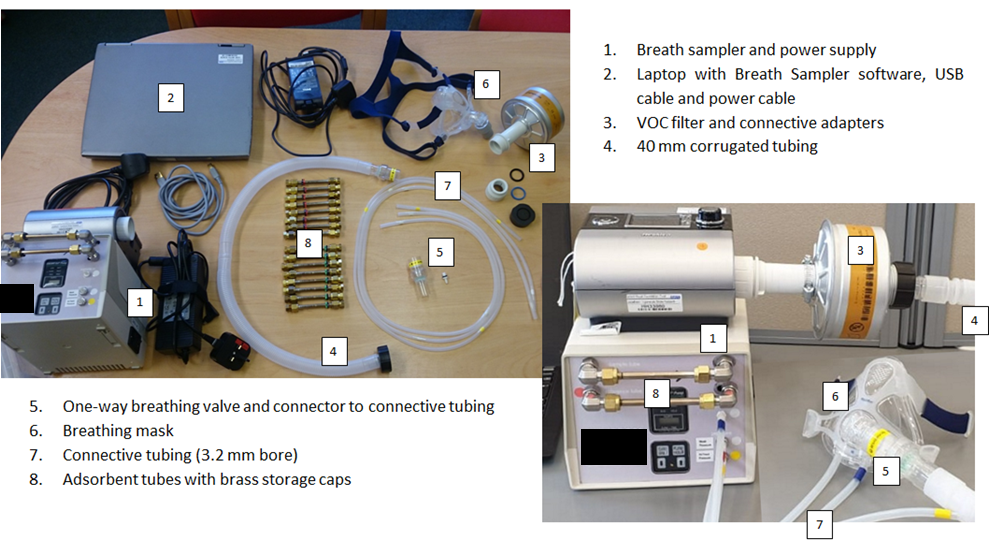
**

**Figure S1. The breath sampling system**


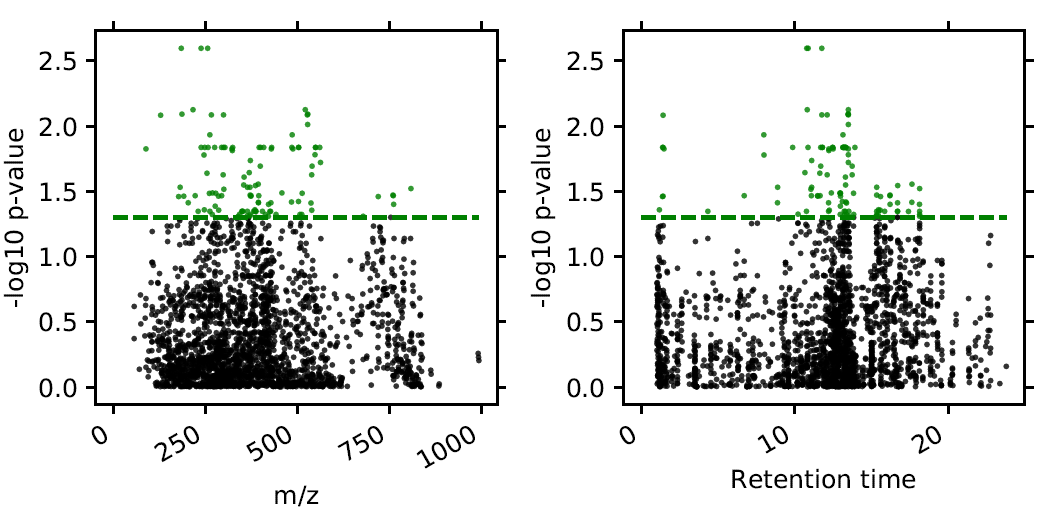


**Figure S2. Manhattan plots highlighting 112/2420 features (green), which showed significance between timepoint groups, selected to build a molecular network for metabolite identification.**


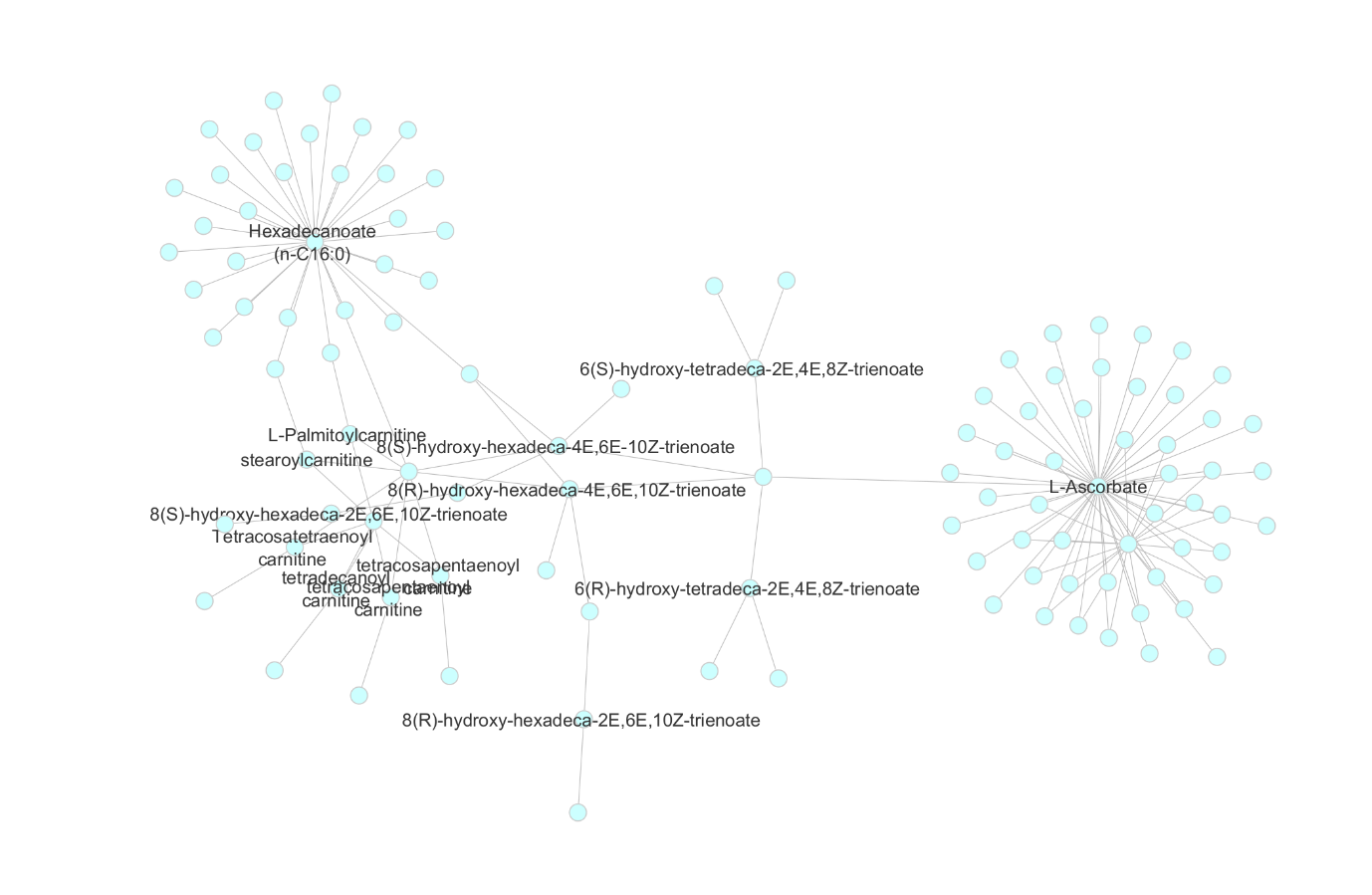


**Figure S3. Molecular network illustrating predicted compounds (nodes) clustered based on similarity to metabolite pathways and other closely related predicted compounds (edges) drawn using *Cytoscape* 3.4.0. Annotated are compound names associated with empirical IDs which display significant differences between timepoint groups. No chiral separation methods were carried out.**
